# GA-YOLOv11: A Lightweight Subway Foreign Object Detection Model Based on Improved YOLOv11

**DOI:** 10.3390/s25196137

**Published:** 2025-10-04

**Authors:** Ning Guo, Min Huang, Wensheng Wang

**Affiliations:** 1 Mechanical Electrical Engineering School, Beijing Information Science and Technology University, Beijing 100192, China; 2023020020@bistu.edu.cn (N.G.); ws_wang@bistu.edu.cn (W.W.); 2 Key Laboratory of Modern Measurement and Control Technology of the Ministry of Education, Beijing Information Science and Technology University, Beijing 100192, China

**Keywords:** foreign body detection, YOLOv11, lightweight module, attention mechanisms

## Abstract

Modern subway platforms are generally equipped with platform screen door systems to enhance safety, but the gap between the platform screen doors and train doors may cause passengers or objects to become trapped, leading to accidents. Addressing the issues of excessive parameter counts and computational complexity in existing foreign object intrusion detection algorithms, as well as false positives and false negatives for small objects, this article introduces a lightweight deep learning model based on YOLOv11n, named GA-YOLOv11. First, a lightweight GhostConv convolution module is introduced into the backbone network to reduce computational resource waste in irrelevant areas, thereby lowering model complexity and computational load. Additionally, the GAM attention mechanism is incorporated into the head network to enhance the model’s ability to distinguish features, enabling precise identification of object location and category, and significantly reducing the probability of false positives and false negatives. Experimental results demonstrate that in comparison to the original YOLOv11n model, the improved model achieves 3.3%, 3.2%, 1.2%, and 3.5% improvements in precision, recall, mAP@0.5, and mAP@0.5: 0.95, respectively. In contrast to the original YOLOv11n model, the number of parameters and GFLOPs were reduced by 18% and 7.9%, respectfully, while maintaining the same model size. The improved model is more lightweight while ensuring real-time performance and accuracy, designed for detecting foreign objects in subway platform gaps.

## 1. Introduction

In urban rail transit systems, platform doors play a crucial role. Not only do they ensure passenger safety and prevent accidents involving passengers falling onto the tracks, but they also improve train operational efficiency. However, foreign objects in the gaps between platform doors, such as items left behind by passengers, trash, or other obstacles, can obstruct the normal closing of platform doors, cause malfunctions, and even lead to serious safety accidents [1,2,3]. Therefore, detecting foreign objects in platform door gaps is extremely important.

In recent years, deep learning technology has achieved remarkable results in the field of object detection, particularly with the use of convolutional neural networks [4]. Representative algorithms include the Region-based Convolutional Neural Network (R-CNN) [5], the Fast Region-based Convolutional Neural Network (Fast R-CNN) [6], Faster R-CNN (Faster Region-based Convolutional Neural Network) [7], and Mask R-CNN [8] and their derivative algorithms have continuously improved average accuracy on public object detection datasets. As the network architecture becomes increasingly complex, the object representations extracted by the model become more abstract. This characteristic enables the model to demonstrate significant performance improvements in both image classification tasks in computer vision and semantic recognition tasks in natural language processing.

Current methods for detecting foreign objects in subway gaps can be broadly categorized into two types: traditional detection approaches and computer image processing-based techniques. Traditional detection primarily involves manual inspections and sensor-based detection methods. Manual inspections consume significant human resources and impose heavy workloads on personnel, while also failing to provide real-time monitoring capabilities.

Currently, methods for detecting foreign objects in subway gaps can be summarized as categorized into two types: traditional detection methods and computer image processing-based methods. Traditional detection methods primarily involve manual inspections and sensor-based detection methods. Manual inspections consume a significant amount of human resources and impose a heavy workload on personnel, while also failing to provide real-time monitoring capabilities. Fei et al. [9] proposed a multi-sensor foreign object detection method that integrates visual cameras and lidar to detect foreign objects. Zhang et al. [10] proposed a complementary infrared and ultrasonic detection method for detecting foreign object intrusion, which can improve detection performance at different distances.

For algorithms used in computer-based foreign object detection, image processing is the primary focus, with deep learning algorithms emerging as the main subject of research. Wu et al. [11] proposed an adaptive moving object detection algorithm based on an improved ViBe algorithm. Zheng et al. [12] utilized machine vision to model the background of subway gaps, assess the degree of feature changes, and thereby detect the presence of foreign objects. Gao et al. [13] developed a railway foreign objects detection algorithm based on the Faster R-CNN network model. This algorithm employs transfer learning techniques to augment railway foreign object intrusion data. However, it may encounter difficulties when identifying foreign object types outside the training dataset and exhibits relatively slow detection speeds. Meanwhile, the detection speed of SSD [14] and YOLO [15] series algorithms has also been significantly improved on public datasets, and target detection algorithms are widely applied across various fields to address detection problems.

Zhang et al. [16] proposed an improved YOLOv7 algorithm for detecting foreign object intrusion on high-speed railways. By incorporating the CARAFE operator, GhostConv convolutions, and a global attention mechanism, this enhanced model achieves a balance between accuracy and efficiency, though computational time increases. Ding et al. [17] increased the model’s recognition capabilities to recognize minute objects in complex environments by integrating YOLOv8 with attention mechanisms. This approach increased computational load and inference time, limiting the model’s applicability on resource-constrained platforms.

Cao et al. [18] redesigned the c2f module in YOLOv8 to reduce model parameter redundancy and further improve detection speed. This method effectively reduces the number of parameters by optimizing the network structure, thereby achieving better performance in terms of speed and resource consumption. However, this parameter compression design reduces the detection accuracy of the model in scenarios where the target distribution is extremely complex or diverse.

As the YOLO series continues to evolve, YOLOv11, the latest iteration, introduces advanced architectural designs and optimization strategies. For example, the C3k2 module, Spatial Pyramid Pooling (SPPF) block, and Spatial Attention (C2PSA) block, among others, these improvements further enhance the model’s feature extraction capabilities and computational efficiency. YOLOv11 maintains high-speed detection while offering higher detection accuracy, making it one of the leading algorithms in the field of object detection [19].

He et al. [20] proposed a multi-scale feature fusion method that significantly enhanced YOLOv11’s detection capability for small objects. However, this approach increased computational costs and reduced inference speed, limiting its application in real-time scenarios. Zhang et al. [21] proposed lightweight convolutional modules. This architecture substantially improved model speed and resource efficiency. However, the lightweight design compromised the model’s ability to represent fine-grained features, resulting in a slight decrease in detection accuracy.

Despite continuous technological advancements, detecting foreign objects in the narrow gap between subway train doors and platform screen doors presents unique challenges: Objects are typically extremely small and susceptible to complex lighting variations such as reflections and shadows. The strict timing requirements for door operations demand both high precision and real-time inference capabilities. Furthermore, the extremely short door opening/closing intervals necessitate ultra-high-speed processing, compelling the system to achieve near-perfect detection reliability with minimal false alarm rates.

Based on the aforementioned issues, this article proposes an improved object detection framework, GA-YOLOv11, tailored for subway safety scenarios. This framework is based on the YOLOv11 architecture, with a focus on optimizing the algorithm to enable real-time detection of foreign objects in the gaps between subway train doors and platform screen doors. First, a dataset containing various categories of foreign objects was constructed and annotated. Subsequently, the YOLOv11 model was specifically optimized and adjusted by integrating the GhostConv network into the backbone network. Additionally, this study introduced the GAM channel attention module before the final convolutional layer in the head network.

The main contributions of this paper are summarized as follows:

1. This study focuses on subway safety scenarios and has constructed a dataset covering multiple types of foreign objects, completing detailed annotation work. The dataset focuses on foreign object detection in the gap between subway train doors and platform screen doors, providing a solid data foundation for subsequent algorithm optimization and model training, and effectively improving the adaptability and accuracy of object detection tasks in practical applications.
2. Based on the YOLOv11n infrastructure, this paper introduces the GhostConv network module, specifically by adding the Ghost module to the backbone network. This module significantly improves network capacity by generating additional feature maps while maintaining low computational costs. This innovative design effectively improves the model’s feature extraction capabilities in an intricate environment, thereby enhancing the efficiencies and performance of foreign object detection.
3. To further enhance the model’s ability to learn the correlations between feature channels, the researchers added a GAM channel attention module before the final convolutional layer of the head network. This module can dynamically adjust the distribution of channel weights, strengthening the model’s ability to capture key features and enabling it to perform better in multi-scale feature representation, thereby improving the accuracy and robustness of real-time detection of foreign objects in subways.

Section 2 provides a detailed description of data collection, the simulation platform, the selection of typical items, and data augmentation. Section 3 introduces the proposed model architecture, including the technical principles behind each module. Section 4 outlines the experimental setup and comparison tests used, followed by a results and comparison analysis. Section 5 concludes the paper with a summary of this study and directions for future work.

## 2. Dataset Preparation

### 2.1. Data Collection

**Figure 1:**
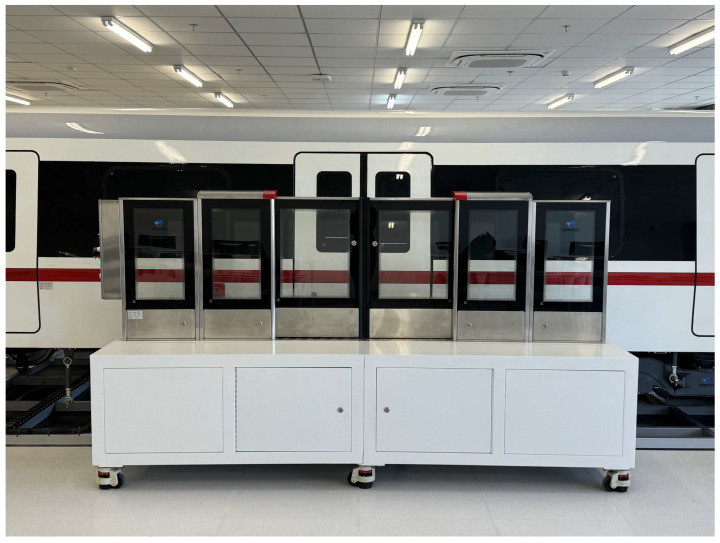
Laboratory data acquisition platform.

In the current study, due to the lack of publicly available standard datasets for detecting foreign objects in subway risk spaces, a subway foreign object dataset was collected and constructed by manually placing foreign object samples in a laboratory simulation environment. The simulation environment is shown in Figure 1.

The parameters of the experimental platform are presented in Table 1.

Following a systematic survey of typical foreign objects that may appear between subway platform doors and train doors, nine representative foreign objects were selected as the subjects for dataset construction. These include: boxes, small knives, badminton rackets, cups, water bottles, tape, sticks, bags, and ropes.

### 2.2. Data Augmentation

**Figure 2:**
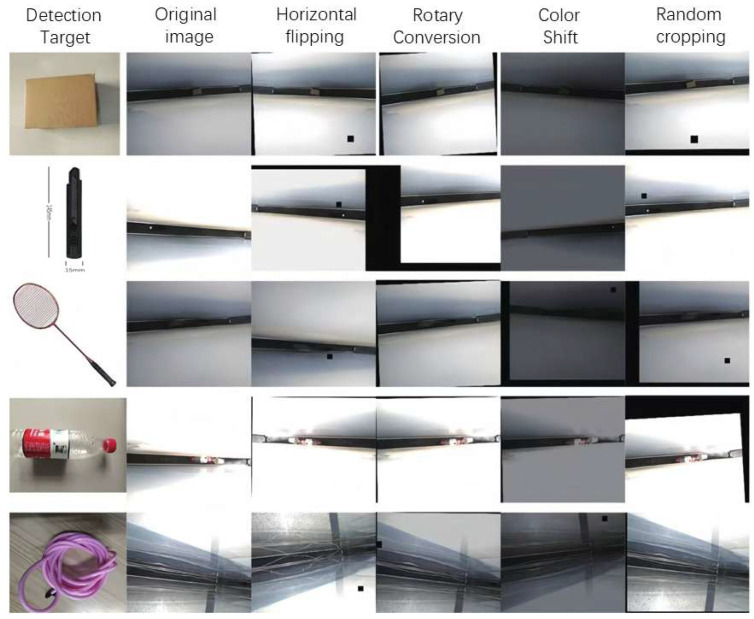
Visual comparison of images before and after data augmentation.

During data collection, complex interference factors in the actual subway operating environment were fully simulated: For underground line scenarios, the simulation focused on image blurring caused by dust accumulation in tunnels and equipment vibration caused by air pressure generated by train operation; for outdoor scenarios, the focus was on simulating complex lighting environments such as changes in sunlight angle, backlighting conditions, and dynamic shadows. To enhance the dataset’s adaptability to different environments, image collection was conducted during different time periods of day and night, resulting in a total of 700 raw video frames. Both figures are adjusted to a uniform resolution of 1280 × 1024 pixels. Furthermore, to enhance sample diversity, this study employs multiple data augmentation techniques [22], which include horizontal flipping and vertical flipping, translation transformations, random cropping, rotation transformations, brightness adjustment, Gaussian noise introduction, and random cropping of local pixel blocks. Through these operations, each original image was expanded into 10 derived samples, ultimately forming a multi-category foreign object detection dataset comprising 7000 images. As shown in Figure 2.

According to the definition provided by the International Society for Optics and Photonics (SPIE), a small target refers to an object in an image whose area is less than 0.12% of the original image area. Statistical analysis reveals that the small target instance in the dataset constructed for this study is “knife”, with pixel dimensions of approximately 23 × 22, accounting for about 0.16% of a 1280 × 1024 resolution image, which is close to the small target threshold criterion. During the data annotation phase, the LabelImg tool was used to annotate the target categories in the sample images, and the annotation results were saved as TXT format files. The specific statistical data is detailed in Table 2. During the data splitting phase, this study used random sampling to divide the expanded dataset into training, validation, and test sets at a ratio of 7:1:2.

## 3. Models and Methods

### 3.1. Original YOLOv11n Model

The YOLOv11 series includes a variety of models, which are primarily categorized into bounding box detection models, instance segmentation models, pose estimation models, rotated bounding box models, and image classification models. Each model also comes in different size variants [23], including Nano (n), Small (s), Medium (m), Large (l), and Extra-Large (x). These models vary in terms of accuracy and speed to accommodate different application requirements. In the YOLOv11 series architecture, while YOLOv11x achieves the highest detection accuracy, its computational complexity and parameter count are significantly higher than other variants, resulting in the slowest inference speed. In contrast, YOLOv11n [24] has the fewest parameters and computational operations, offering a lightweight advantage for fast real-time detection, though accuracy is correspondingly reduced. Considering the resource constraints of subway embedded devices, where hardware platform computational capabilities and memory space are limited, and given that this study aims to achieve rapid real-time detection of foreign objects between subway platform doors and train doors in a subway environment, this paper selects YOLOv11n, which offers the optimal computational efficiency, as the base model.

**Figure 3:**
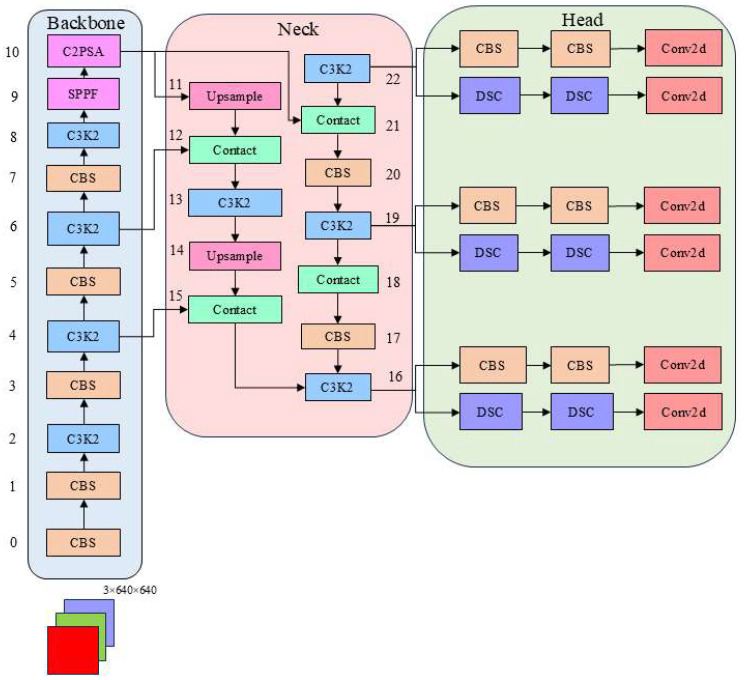
YOLOv11n network model structure.

The network structure of YOLOv11n consists of three main components: the backbone network (Backbone), the neck network (Neck), and the detection head (Head), as shown in Figure 3.

The backbone section introduces C3k2 blocks to handle feature extraction at different stages of the backbone. It employs two smaller convolutional kernels instead of a single large convolutional kernel. YOLOv11 keeps the SPPF blocks from previous editions and presents a new cross-stage section, the C2PSA block. Through spatial feature pooling, the C2PSA component enables YOLOv11 to focus detection on specific regions, which is expected to enhance recognition accuracy for objects of varying sizes and locations [25]. This neck module integrates diverse dimensional feature data and outputs the processed information to the head module to assist in subsequent predictive tasks. This process often requires upsampling operations on feature maps from different levels and feature map concatenation and fusion, which helps the model efficiently capture multi-scale feature information. The head network bears the responsibility of producing the final prediction results, encompassing two tasks: target identification and category classification. By deeply processing the feature maps passed from the neck, the head ultimately outputs the bounding box coordinates and category label information of objects in the image.

Overall, YOLOv11n is a lightweight and high-precision object detection model. This article selects this network and further optimizes and improves it.

### 3.2. Improved Network Model

**Figure 4:**
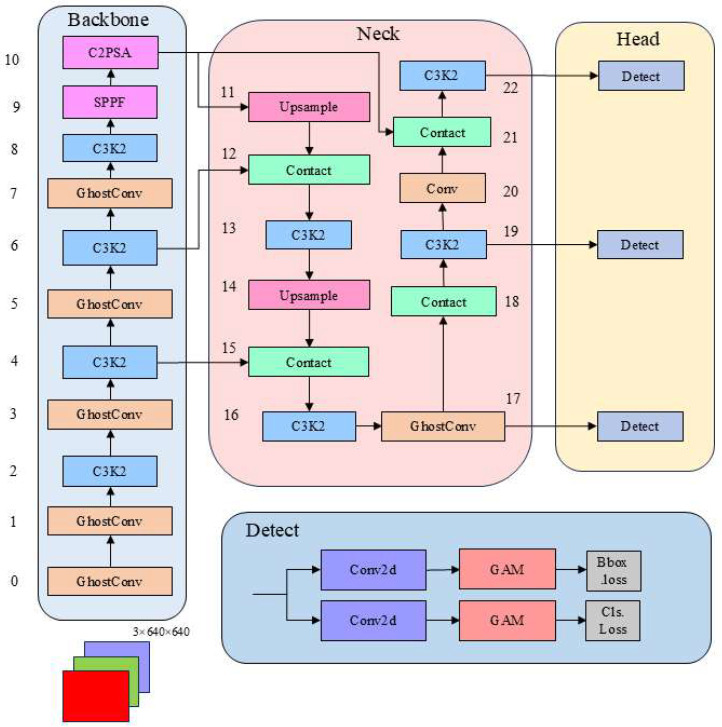
GA-YOLOv11 network model structure.

To address the issues of difficult model deployment, low detection accuracy, and high false positive rates in detecting foreign objects in the gap between subway train doors and platform screen doors, this article presents an optimized detection architecture for the YOLOv11 algorithm, named GA-YOLOv11; its network architecture is shown in Figure 4.

This model aims to address issues such as low accuracy, excessive parameter counts, and large model sizes in traditional networks used for foreign object detection in urban subways. The model includes the following two improvements to optimize performance. The introduction of lightweight GhostConv convolution modules in the backbone network reduces computational resource waste in irrelevant areas. Significantly enhance the ability to distinguish the attributes of various miniature targets. This improves the accuracy of small target detection, reduces model complexity and computational load, enabling YOLOv11 to construct a more lightweight network architecture. The introduction of the GAM attention mechanism in the head network helps the model more accurately distinguish the location and category of objects, reducing misclassifications and false negatives. This effectively suppresses interference from complex backgrounds, minimizes information loss, and improved the model’s robustness and accuracy in the detection of foreign objects in subway gaps.

#### 3.2.1. Ghost Network

**Figure 5:**
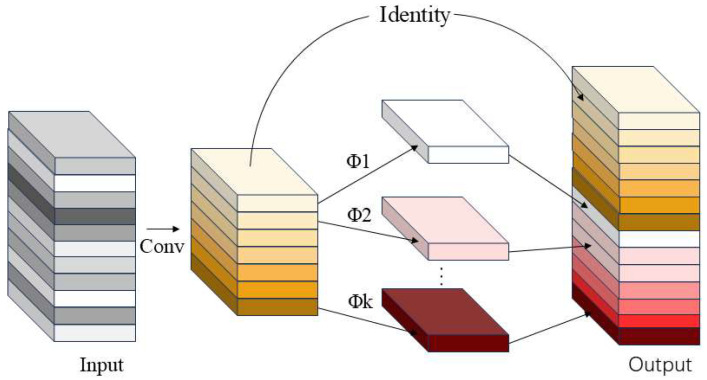
The module structure of GhostNet.

GhostNet is a lightweight network [26] with a structure shown in Figure 5. GhostNet achieves this goal by introducing a new type of convolution module called GhostConv [27]. As a lightweight convolutional module, GhostConv is designed to replace the fully connected layer of conventional convolutional layers. By optimizing the computational process of convolutional layers, it effectively reduces computational complexity while maintaining the output feature map size and channel count, ensuring model performance, and significantly reducing computational load and parameter count. Specifically, the computational method adopted by the GhostConv module is referred to as a “cheap operation,” which is characterized by its simplicity and low computational cost.

As Figure 5 shows, the processing flow encompasses three steps: generating the main part of the feature map in the initial stage, then using the convolution kernel to obtain the remaining features through low-cost, inexpensive calculations, and finally integrating and processing the two parts of features generated in the previous and final stages.

Specific steps: First step: Use traditional convolution operations to obtain the internal feature map $Y^{\prime }$. When the bias term is ignored, the computational complexity of this section is approximately equal to:

(1) $$Y^{\prime }=X*f$$

Among these, Y′ carries dual significance: functionally, it serves as the “intrinsic feature map” extracted through a single costly convolution operation, forming the foundation for subsequent feature generation; computationally, this convolution operation constitutes the module’s “primary computational overhead” due to its substantial parameter count and computational demands. Thus, Y′ represents both the core of the information flow and the primary manifestation of computational complexity.

Then, use operation $\Phi_{i,j}$ to generate ghost feature maps $y_{ij}$ from the feature maps of each channel of $Y^{\prime }$.

(2) $$y_{ij}=\Phi_{i,j}(y_{i}^{\prime }),\forall i=1,\ldots ,m,j=1,\ldots ,s$$

In this equation, $y_{i}^{\prime }$ denotes the initial feature map of the i-th item, Under the action of the linear transformation $\Phi_{i,j}$, it evolves into the so-called “Ghost” feature map $y_{ij}$ of the j-th item. Each initial feature map ${\left.\left\{y_{ij}\right.\right\}}_{j=1}^{s}$ can generate s corresponding “Ghost” feature maps $y_{ij}$. Throughout this process, the $\Phi_{i,s}$ function ultimately manifests as an identity mapping, whose role is to preserve the integrity of the initial feature maps. Finally, the final result Output is obtained by connecting the essential attribute map extracted during the initial phase and the “Ghost” feature map acquired in the next step through an identity mapping.

**Figure 6:**
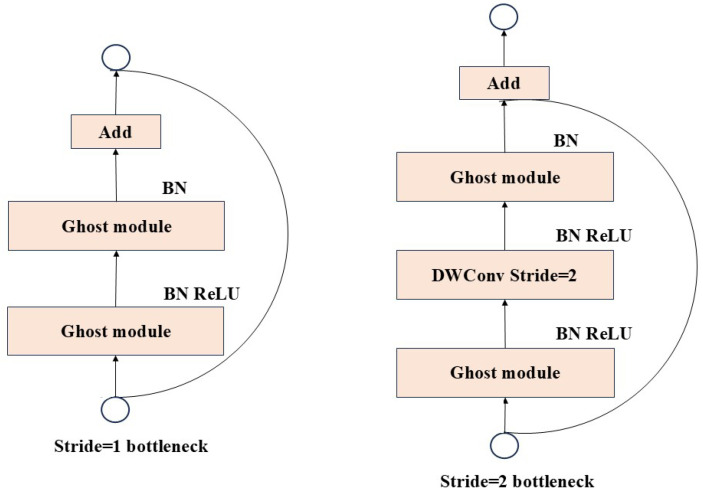
Ghost Bottleneck Network Bottleneck Structure.

Figure 6 illustrates that the process of addressing the Ghost Bottleneck involves: After the first GhostConv generates two feature maps, batch normalization and ReLU activation function operations are performed successively. Next, perform the second GhostConv for convolution processing, and finally output the feature map through an Add operation.

This Ghost module features identity mapping capabilities and linear processing of $m\cdot \left(s-1\right)=\frac{n}{s}\cdot \left(s-1\right)$ lines, with a core average scale of d×d for each linear processing operation. Each linear operation can have different shapes and parameters, but considering the practicality of CPUs or GPUs, this can hinder online inference. Therefore, to achieve efficient hardware implementation, it is recommended that each linear operation in the Ghost module use linear operations of the same size.

After optimizing the conventional convolution algorithm using the Ghost module, the theoretical acceleration ratio $r_{s}$ has been improved to:

(3) $$r_{s}=\frac{n\cdot h^{\prime }\cdot w^{\prime }\cdot c\cdot k\cdot k}{\frac{n}{s}\cdot h^{\prime }\cdot w^{\prime }\cdot c\cdot k\cdot k+\left(s-1\right)\cdot \frac{n}{s}\cdot h^{\prime }\cdot w^{\prime }\cdot d\cdot d}=\frac{c\cdot k\cdot k}{\frac{1}{s}\cdot c\cdot k\cdot k+\frac{\left(s-1\right)}{s}\cdot d\cdot d}\approx \frac{s\cdot c}{s+c-1}\approx s$$

In the formula, n represents the input batch size, h′ and w′ denote the height and width of the output feature map, respectively, c represents the number of output channels, k is the size of the convolutional kernel, s is the number of features generated by the main convolution in the Ghost module, and d represents the kernel size for depthwise convolution. Typically, the size of the depthwise convolutional kernel generated by the ghost feature in the Ghost module is identical to k. The scale of the d×d parameter is similar to that of k×k and s≪c. Similarly, the compression ratio $r_{c}$ can be derived using the following formula:

(4) $$r_{c}=\frac{n\cdot c\cdot k\cdot k}{\frac{n}{s}\cdot c\cdot k\cdot k+\left(s-1\right)\cdot \frac{n}{s}\cdot d\cdot d}\approx \frac{s\cdot c}{s+c-1}\approx \frac{s\cdot c}{c}=s$$

If the output channel values of c increase, the mathematical expressions for parameter compression ratio and theoretical acceleration ratio converge to very similar or even identical forms. The Ghost module generates the vast majority of ghost feature maps using low-cost linear operations, resulting in highly consistent and predictable efficiency improvements in both parameter reduction and computational load reduction. The theoretical acceleration ratio is directly proportional to the parameter compression ratio.

When the Ghost component is integrated into the core architecture, learning efficiency is enhanced. By generating “Ghost” feature maps, the model has achieved a significant reduction in the resources required for processing, while also decreasing the scale of model parameters and computational complexity.

#### 3.2.2. GAM Module

**Figure 7:**
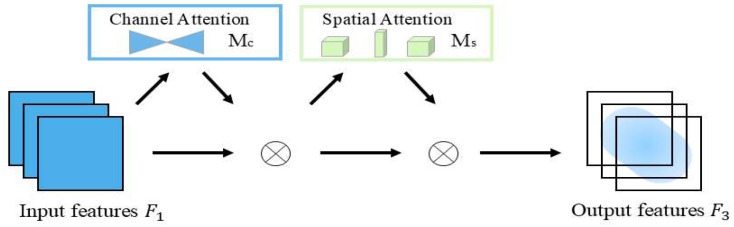
GAM module structure.

GAM (Global Attention Mechanism) is an attention architecture applied in the field of natural language processing [28]. This mechanism aims to enhance the model’s ability to process long text sequences. Traditional attention mechanisms may struggle when handling long texts because they only focus on local contextual information. GAM introduces a global attention distribution to better capture key information across the entire text sequence. The core idea of GAM is that each word or position has different importance in the original attention mechanism [29]. In traditional attention mechanisms, the attention distribution is calculated based on the relative positions between words. In GAM, the global attention distribution is calculated using global information, enabling the model to better understand the entire text sequence. Figure 7 illustrates the structural composition of GAM.

Given the input feature map $F_{1}$, transition feature map $F_{2}$, and output feature map $F_{3}$, the processing flow proceeds as follows: First, perform a channel-by-channel multiplication between $F_{1}$ and the channel attention weight map $M_{c}$ to amplify the responses of channels contributing to the task. Subsequently, multiply the channel-modulated feature map element-wise with the spatial attention map $M_{s}$ to further enhance the representation of key regions in spatial positions.

**Figure 8:**
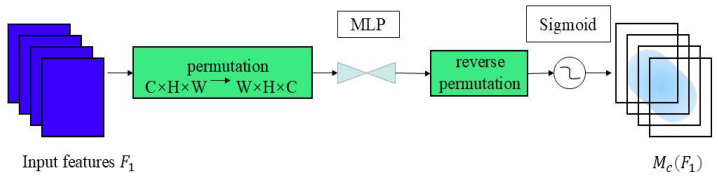
Channel attention sub-module.

Figure 8 illustrates the channel attention sub-module. The input feature map $F_{1}$ comprises specific dimensions in the format C×H×W, where C denotes the number of channels, the H represents the height of the feature map, and the W denotes its width. This submodule performs a dimensional transformation on the feature map using a 3D permutation strategy, converting the original three-dimensional structure from C×H×W to W×H×C to achieve efficient storage and interaction of information. Based on this, a nonlinear mapping relationship between the channel and spatial dimensions is constructed using to enhance the representational power of features through a multilayer perceptron (MLP) network with a dual-layer structure. Given the input feature map $F_{1}\in RC\times H\times W$, the intermediate state feature map $F_{2}$ and output feature map $F_{3}$ are defined as:

(5) $$F_{2}=M_{c}\left(F_{1}\right)⊗F_{1}$$

(6) $$F_{3}=M_{s}\left(F_{2}\right)⊗F_{2}$$

In the equation, $M_{c}$ and $M_{s}$ represent the channel and spatial attention maps, respectively, ⊗ denotes element-wise multiplication.

**Figure 9:**
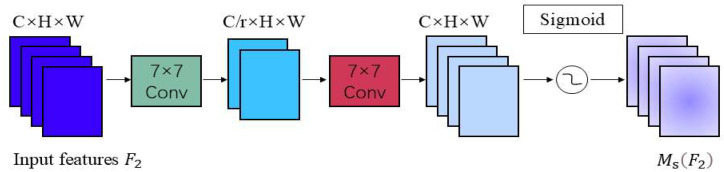
Spatial attention sub-module.

As shown in Figure 9, the spatial attention sub-module enhances the model’s capability to capture spatial information by highlighting key spatial locations within feature maps. To ensure consistency with the channel attention module, both modules use the same reduction ratio. To focus on spatial information, the spatial attention module integrates spatial feature information from the input map through two layers of 7 × 7 convolutions. The input feature map $F_{2}$ has dimensions C×H×W. First, feature extraction is performed using a 7 × 7 convolution layer, with the output dimensions of the convolution layer being C/r×H×W, where r is the reduction ratio used to reduce the number of parameters and computational complexity. Next, another 7 × 7 convolution layer is used to further fuse spatial information, with the output dimensions restored to C×H×W.

## 4. Experiments

### 4.1. Experimental Configuration and Training Parameters

See Table 3 and Table 4 for detailed specifications of the hardware and software configurations required for the experiment.

To ensure the optimal configuration of the experimental parameters, we supplemented the experiments in the Appendix A and came up with the following pairings as the best parameter choices for the experiments.

### 4.2. Evaluation Indicators

In evaluating model performance, this study adopted a series of key metrics, including Precision, Recall, Mean Average Precision of 50% (mAP@0.5), Mean Average Precision of 50–95% (mAP@0.5:0.95), Frames Per Second (FPS), Parameters (Params), billion Floating Point Operations (GFLOPs), and Model Size. The specific formulas are as follows:

(7) $$Precision=\frac{TP}{TP+FP}$$

(8) $$Recall=\frac{TP}{TP+FN}$$

In Equations (7) and (8), TP represents the number of actual positive samples correctly identified as positive by the model, FP denotes the number of actual negative samples incorrectly classified as positive, and FN indicates the number of actual positive samples incorrectly classified as negative. The formula for calculating average accuracy can be expressed as:

(9) $$AP=\int_{0}^{1}P(R)dR$$

(10) $$mAP=\frac{\sum AP}{n}$$

Among them, n denotes the number of categories. mAP represents the mean average precision across all detected categories. mAP@0.5 evaluates model performance when the Intersection over Union (IoU) between predicted bounding boxes and ground truth bounding boxes is 0.5; mAP@0.5:0.95, meanwhile, refers to the mean average precision that the model can achieve when the IoU threshold ranges from 0.5 to 0.95.

### 4.3. Model Training Experiment Results

The detection results of the improved model are shown in Table 5.

To validate the behavior of the GA-YOLOv11 algorithm, the model was compared in terms of performance with YOLOv11n and other network models, including DeeplabV3-CSPNet, U-Net model, YOLOv7s, YOLOv8n, and YOLOv10n. Table 6 shows the comparison results of key detection algorithm.

The analysis of experimental results yields the following conclusion: The model proposed in this article outperforms all comparison models in mAP@0.5, achieving an average precision value of 99.3%, which is a 1.2% improvement over the original YOLOv11n model. Notably, while improving mAP, the model’s memory footprint remains unchanged (both at 5.35), and the improved model’s FPS shows a significant increase compared to the original YOLOv11n (from 21.42 to 23.65). Considering both FPS and memory footprint, the improvements proposed in this paper demonstrate their effectiveness.

Overall, the proposed model achieves optimal performance across three key metrics:

1. The mAP metric achieved a peak value of 99.3%, surpassing all other models considered in the comparative analysis;
2. The FPS metric demonstrated the most outstanding performance among all comparison models, reaching 23.65 frames per second, outperforming all other models evaluated in the comparative study;
3. The model occupies the smallest space at 5.35 MB, demonstrating outstanding performance surpassing all other models assessed in the comparative analysis.

The optimization has yielded tangible results, with the adjusted model successfully balancing portability while significantly enhancing detection accuracy and processing efficiency. This comprehensive advantage in terms of accuracy, speed, and resource utilization fully validates the effectiveness and advanced nature of the improved model in practical engineering applications; this makes it ideal for real-time detection of foreign objects in subway train door gaps.

### 4.4. Ablation Experiment Results

In order to systematically evaluation of the effectiveness of individual improvement modules in the GA-YOLOv11 model in the object detection task on the subway train door gap foreign object dataset, this section conducted ablation analysis experiments. Using YOLOv11n as the baseline model and keeping all parameters consistent, the GhostConv module and GAM attention mechanism were gradually introduced. The results are shown to the table below, where “√” represents for this module has been added, while “×” represents for this module has not been added. A total of four fading experiments have been conducted. All fading experiments employed the identical dataset and hardware/software configuration. Other metrics such as mAP and FPS obtained from the model detection results are shown in Table 7.

The results of the ablation experiments are shown in Table 7. It is observed that in comparison to the original YOLOv11n, when the GhostNet module is introduced, although the precision and recall rate decrease by 0.3% and 0.2%, the mAP@0.5 and mAP@0.5:0.95 increase by 0.2% and 1.0%, respectively, and reduction in the number of parameters by 18.0%. When only the GAM attention mechanism is introduced, although the detection speed slows down, the precision, recall, mAP@0.5 and mAP@0.5:0.95 increase by 2.0%, 1.2%, 1.3% and 1.6%, respectively, and the detection speed is 21.22 frames per second. When both the GhostNet module and the GAM attention mechanism are introduced, the model improvement effect is the most significant, with the precision rate reaching 98.4%, an increase of 3.3%; the recall rate increases by 3.2%; mAP@0.5 is 99.3%, an increase of 1.2%; mAP@0.5:0.95 is 86.9%, an increase of 3.5%; the parameter count was reduced by 18%, achieving a 23.65 frames per second detection speed.

**Figure 10:**
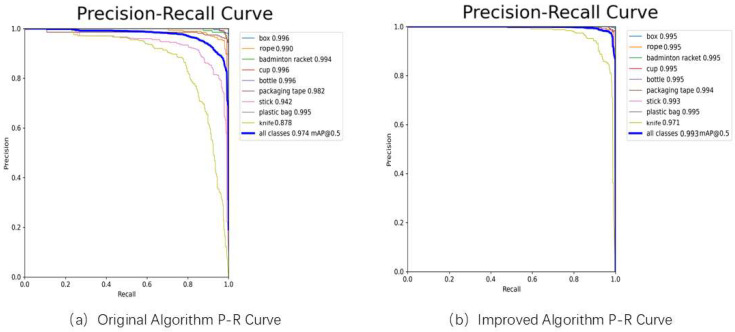
Comparison of P-R Curves Between the Original Algorithm and the Improved Algorithm.

Figure 10 presents a comparison of the Precision-Recall curves for the original and improved algorithms in the object detection task. The improved algorithm demonstrates higher precision across all categories, with particularly significant gains in the small object “knife” category, rising from 87.8% to 97.1%. The algorithm’s mAP@0.5 improved from 97.4% to 99.3%, indicating that the enhancement measures effectively boosted the model’s detection accuracy and robustness, this further validates that the effectiveness for the proposed method.

**Figure 11:**
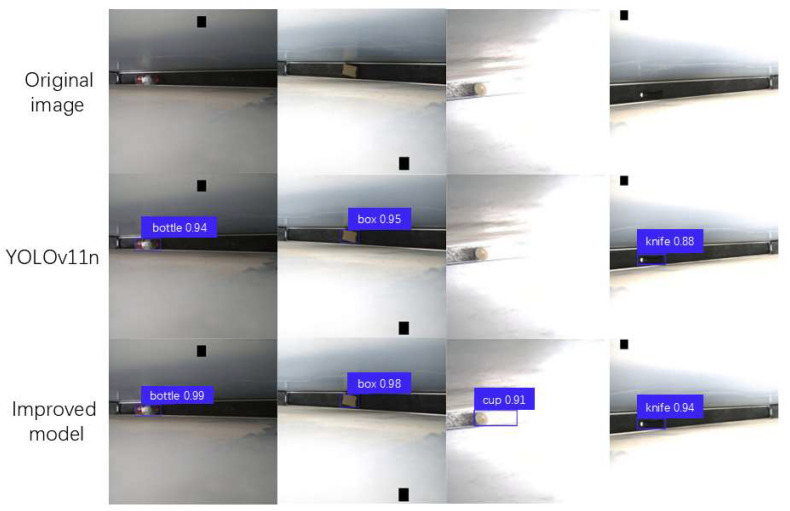
Comparison of object detection performance before and after model improvement.

Experimental results demonstrate the improvement that the enhanced GA-YOLOv11 model can not only boost the accuracy of object detection with the model, but also achieve model lightweighting, meeting the requirements of the subway foreign object detection system in terms of detection accuracy and real-time performance. Figure 11 compares the foreign object detection performance before and after the model improvement. Observably, the original YOLOv11n model still has some omissions from foreign object detection, for instance, failing to detect foreign objects under strong light situations. The original YOLOv11n model also lacks the high confidence levels of the improved model in scenarios involving small target detection, low light conditions, and normal lighting.

**Figure 12:**
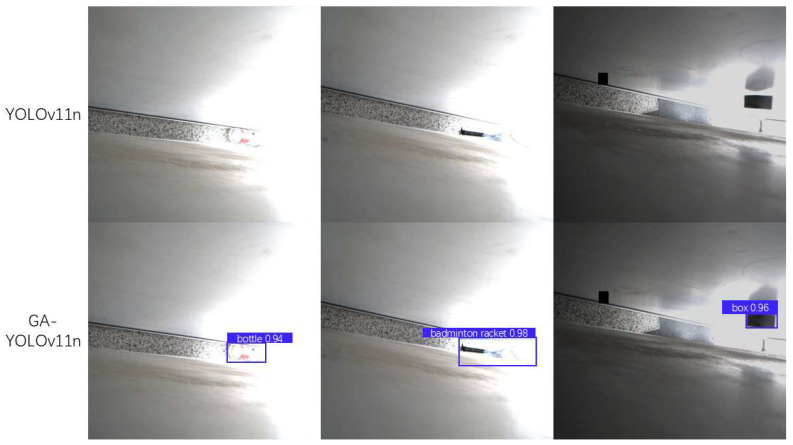
Comparison of detection results in strong light environments.

We observed a significant improvement in the enhanced GA-YOLOv11 model’s ability to recognize objects under strong light conditions. To further validate this finding, we extracted a “strong light subset” from the original dataset and conducted systematic testing on it. As shown in Figure 12, the enhanced GA-YOLOv11 model demonstrates markedly improved robustness against strong light. Results indicate that the original YOLOv11n model exhibits noticeable false negatives under strong light interference, failing to effectively detect multiple objects. In contrast, GA-YOLOv11n not only substantially reduces false negatives but also achieves more accurate object localization and recognition, demonstrating superior lighting invariance. As illustrated, in identical high-light scenarios, the improved model successfully detected multiple objects with high confidence, whereas the original model failed to detect some clearly visible targets. This comparison visually demonstrates the practicality and effectiveness of our approach under complex lighting conditions.

**Figure 13:**
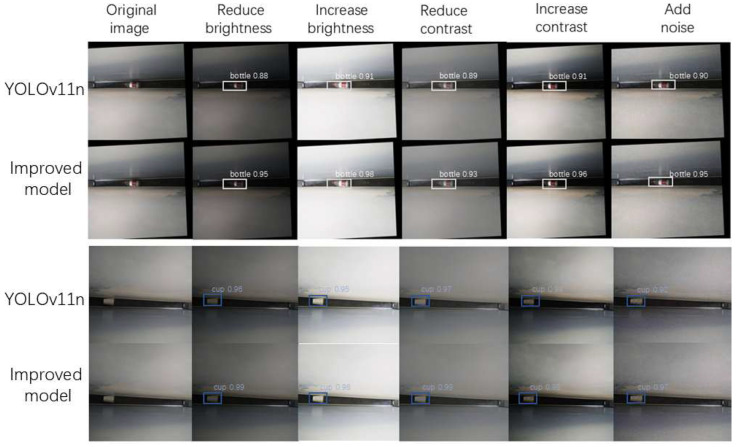
Comparison of object detection performance before and after model refinement under varying brightness, contrast, and noise levels.

To provide a more intuitive comparison of the detection differences between the improved model and the baseline model, we tested the results under varying brightness/contrast/noise levels in the test images, as shown in Figure 13. The table shows image annotations as category probabilities.

In summary, the GA-YOLOv11 model not only effectively mitigates the false negative issue of the original model in scenes with strong light interference but also achieves higher detection success rates when dealing with real-world scenarios involving lighting variations and noise pollution. Which gives additional confirmation of the usefulness and practical value to the improvement strategy adopted in this article.

### 4.5. Generalization Experiment Results

**Figure 14:**
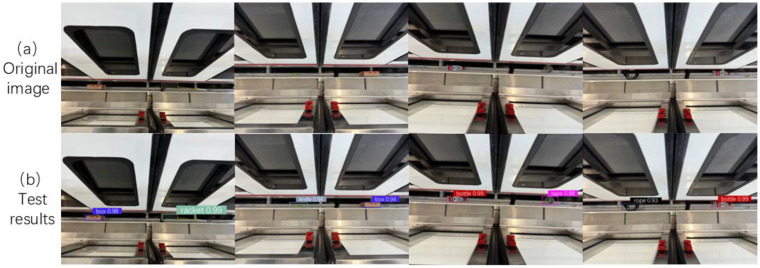
A comparison of object detection performance before and after model refinement.

**Figure 15:**
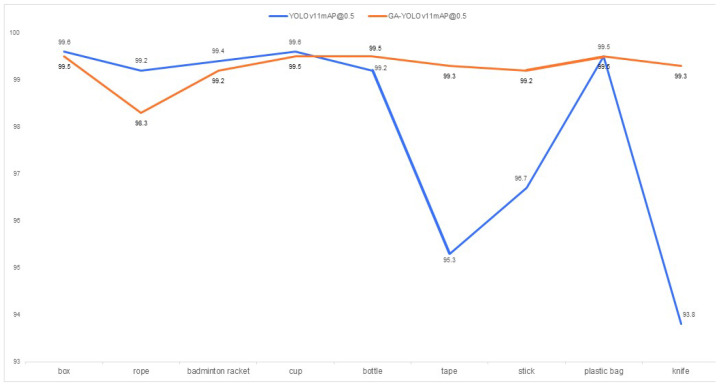
Comparison of mAP@0.5 metrics across categories between the original and improved models in the generalization experiment.

In order to provide further evidence of the algorithm’s generalization capability and robustness, the training dataset used in the experiment was a foreign object dataset collected from a laboratory platform, while the generalization experiment dataset used 500 foreign object images collected from a real environment, including random combinations of different objects in different positions. The camera model and position were kept consistent with the conditions during dataset collection. The annotation process also utilized the LabelImg tool to label the target categories in the sample images, with annotation results saved as TXT format files. Figure 14a displays partial sample images, with experimental results illustrated in Figure 14b:

As illustrated by Figure 15, experimental data from the generalized test set indicates that the GA-YOLOv11 model demonstrates a superior level of robustness across nine categories compared to the essentially baseline YOLOv11 model. Specifically, the “knife” category has been elevated by 5.5% (93.8% to 99.3%), the “tape” category by 4.0% (95.3% to 99.3%), and the “stick” category by 2.5% (96.7% to 99.2%). Concurrently, the “box” and “bottle” categories also achieved performance gains of 0.1% and 0.3%, respectively.

Under identical conditions, the generalization experiment results are shown in Table 8. It is evident that the proposed improved algorithm demonstrates significantly superior generalization performance compared to the original algorithm, achieving an accuracy improvement of 3.3%. In addition, the algorithm improved the mAP value by 1.2% in recognizing foreign object types.

## 5. Conclusions

This article proposes an efficient, lightweight genetic algorithm-based YOLOv11 network model for detecting and characterizing foreign objects inside subway train door gaps, based on an enhanced YOLOv11 framework. Addressing the issues of false positives and false negatives, as well as the original model’s large number of model parameters, this model introduces the GhostConv module, which focuses on decreasing the amount of parameters and the calculation loading, reducing inference latency, and improving system response speed. Additionally, to enhance feature fusion and improve focus on small targets, the improved model incorporates the GAM module. This model achieves improvements of 3.3%, 3.2%, 1.2%, and 3.5% in precision, recall, mAP@0.5, and mAP@0.5:0.95, respectively. The number of parameters and GFLOPs are reduced by 18% and 7.9%, respectively, compared to the original YOLOv11n model, while maintaining the same model size. This paper focuses on optimizing network architecture, innovating algorithms, and exploring the design structure of new models to significantly reduce the number of parameters and the complexity of calculations while maintaining high accuracy, thereby achieving the goal of network model lightweighting. The enhanced model improves the ability to capture foreign object features in subway environments, particularly when foreign objects are similar to the background or under complex lighting conditions, thereby enhancing detection accuracy in subway platform gap foreign object detection scenarios.

However, the model still exhibits certain limitations. Although the integration of GhostConv with the GAM module significantly reduces computational costs and enhances detection performance, the model’s accuracy may slightly decrease when dealing with foreign objects that are highly irregular in shape or have extremely low background contrast. Furthermore, the current model optimization primarily targets common foreign object categories, and its performance on rare or unseen target types requires further validation.

With the continuous development of urban rail transit, the importance of automatic detection of foreign objects between subway platform doors and train doors has become increasingly prominent. The algorithm model proposed in this paper holds broad application prospects. Future research will focus on collecting foreign object intrusion data in real subway platform environments to construct a large-scale sample space, thereby enhancing the algorithm’s accuracy and robustness. Concurrently, research will also strive to conduct large-scale field testing in actual subway environments to validate and optimize the model’s performance under real-world operational conditions.

## Figures and Tables

**Table 1:** Parameters of experimental platform.

Name	Specifications
Platform door	1.5 m × 2 m
Train door	1.85 m × 2 m
Platform gap	10 cm
Camera	Hikvision Industrial Camera MV-CU013-A0UC (Hangzhou Hikvision Digital Technology Co., Ltd., located in Hangzhou, China)

**Table 2:** Dataset statistics.

Object Category	Training Set	Validation Set	Test Set	Total
box	525	75	152	752
knife	564	82	264	910
badminton racket	508	74	149	731
cup	476	68	132	676
bottle	602	86	172	860
tape	543	79	158	780
stick	609	87	174	870
plastic bag	466	68	136	670
rope	522	76	153	751
Total	4815	695	1490	7000

**Table 3:** Experimental platform configuration parameters.

Device	Configuration
System	Windows 11
Pytorch	2.4
Python	3.10
Cuda	11.8
CPU	13th Gen Intel(R) Core(TM) i9-13900H (Intel Corporation, Santa Clara, CA, USA)
GPU	NVIDIA GeForce RTX 4060 Laptop GPU (NVIDIA Corporation, Santa Clara, CA, USA)

**Table 4:** Parameter Settings for the Experimental Platform.

Parameter	Setting
Input image size	640 × 640
Epochs	160
Batch size	8
Initial learning rate	0.01
Optimizer	Adam
Scale factor	1.2

**Table 5:** Experimental related indicators.

Object Category	Precision (%)	Recall (%)	mAP@0.5 (%)	mAP@0.5:0.95 (%)
box	98.3	100	99.5	84.3
knife	97.7	99.0	99.1	83.2
badminton racket	99.7	100	99.2	88.8
cup	99.9	97.8	99.5	86.4
bottle	99.7	100	99.5	88.8
tape	98.4	99.4	99.2	84.4
stick	98.3	98.6	99.2	87.6
plastic bag	98.3	100	99.5	93.4
rope	93.8	95.7	98.3	85.2
Average	98.4	98.8	99.3	86.9

**Table 6:** Comparison of experimental results for different network models.

Model	mAP @0.5 (%)	Params (M)	Size (MB)	GFLOPs	FPS
DeeplabV3-CSPNet	81.1	41.30	157.3	7.80	12.08
U-Net	83.1	34.60	49.0	7.76	11.29
YOLOv7s	92.2	23.30	184.0	38.0	17.63
YOLOv8n	84.1	21.67	172.0	59.5	13.38
YOLOv10n	97.5	24.80	59.8	40.5	10.42
YOLOv11n	98.1	25.84	5.35	6.3	21.42
GA-YOLOv11	**99.3**	**21.17**	**5.35**	**5.8**	**23.65**

The bold part is the best value.

**Table 7:** Results of model ablation experiment.

GhostNet	GAM	Precision (%)	Recall (%)	mAP @0.5 (%)	mAP@0.5:0.95 (%)	Params (M)	FPS
×	×	95.1	95.6	98.1	83.4	25.84	21.42 (±0.4)
√	×	94.8	95.4	98.3	84.4	21.19	25.70 (±0.1)
×	√	97.1	96.8	99.4	85.0	23.72	21.22 (±0.2)
√	√	98.4	98.8	99.3	86.9	21.17	23.65 (±0.1)

All indicators in the table are reported as mean ± standard deviation.

**Table 8:** Comparison of different models’ performance in generalization experiments.

Model	Precision (%)	mAP (%)
YOLOv11	95.1	98.1
GA-YOLOv11	98.4	99.3

## Data Availability

The data presented in this study are not publicly available due to privacy and confidentiality restrictions.

## Appendix A

In this appendix, we trained the GA-YOLOv11 model on the laboratory dataset for 160 epochs using the Adam optimizer. To determine the optimal training configuration, we investigated the impact of different learning rate (LR) and batch size (BS) combinations on training performance.

Table A1 summarizes the accuracy and training time for each LR-BS combination. The learning rate significantly impacts accuracy: when LR = 0.001, accuracy falls below 90%; increasing LR to 0.01 resulted in accuracy exceeding 99%. BS primarily affects computational efficiency. Increasing BS from 4 to 8 nearly halves training time, while further increasing to 16 yields only marginal savings. Based on comprehensive training results, we select LR = 0.01 and BS = 8 as the optimal configuration.

**Table A1:** Impact of Different Configurations on Training Performance.

Configuration	Precision (%)	Training Time (h)
LR = 0.001, BS = 4	78.8	4.91
LR = 0.01, BS = 4	92.7	4.46
LR = 0.001, BS = 8	89.5	2.27
LR = 0.01, BS = 8	**99.3**	**2.28**
LR = 0.001, BS = 16	83.9	2.22
LR = 0.01, BS = 16	97.7	2.23

The bold part is the best value.
